# Molecular docking analysis of syringic acid with proteins in inflammatory cascade

**DOI:** 10.6026/97320630018219

**Published:** 2022-03-31

**Authors:** Gheena Sukumaran, Devaraj Ezhilarasan, Pratibha Ramani, R Jancy Merlin

**Affiliations:** 1 Department of Oral Pathology, Saveetha Dental College and Hospitals, Saveetha Institute of Medical and Technical Sciences, Chennai, Tamil Nadu, India; 2 Department of Pharmacology,Biomedical Research Unit and Laboratory Animal Centre, Saveetha Dental College, Saveetha Institute of Medical and Technical Sciences, Chennai, Tamil Nadu, India; 3 Department of Oral Pathology, Saveetha Dental College and Hospitals, Saveetha Institute of Medical and Technical Sciences, Chennai, Tamil Nadu, India; 4 Department of Advanced Zoology and Biotechnology, Women’s Christian College, Chennai, Tamil Nadu, India

**Keywords:** NF kappa b, P50, NF kappa b P65, receptor ligands tnf, Syringic acid

## Abstract

Syringic Acid (SA) is a dimethoxybenzene derived from plants. Dietary SA possesses anti-obesity, anti-inflammatory and anti-steatotic effects and is of interest as a potential therapeutic medication in the treatment of obesity, diabetes, diabetic cataracts and asthma. It has anti-tumorigenic effect against hepatocellular carcinoma, lung carcinoma and oral mucosal carcinoma. It is also believed to have a protective effect on Acetaminophen induced damage in Wistar rats. Therefore, it is of interest to document the molecular docking analysis of syringic acid with proteins in inflammatory cascade such as TNF α, NFκB, P50, P65 and IKB for further consideration in drug discovery.

## Background:

Syringic Acid (SA) is a dimethoxybenzene. It is a 4-hydroxy-3, 5-dimethoxybenzoic acid. It is a metabolite derived from plants and is a conjugate acid of a syringate [1]. Dietary SA possesses anti-obesity, anti-inflammatory and anti-steatotic effects may be due to the regulation of the various metabolic pathways. SA has been touted to have the potential to be a neo therapeutic agent for obesity and non- alcoholic liver disease [2]. A study demonstrated that SA has a protective effect on rats by lieu of the rats not developing experimentally induced diabetes cataracts both in vitro and in vivo [3]. SA is known to have multiple effects in mitigating Reperfusion injuries in case of infarcts. In the case of myocardial ischemic reperfusion injury, SA mediates its effect by its anti- apoptotic property via p13K/Akt/GSK-3β signaling. It is crucial to conferring cardio protection. Activated Akt causes phosphorylation of GSK-3β initiating the translocation to mitochondria with inhibition of the mitochondrial permeability transition pore opening thus salvaging mitochondrial induced apoptosis [4]. SA has a beneficial effect on asthma thought to be due to its interaction with inflammatory cells, inflammatory and antioxidant markers in an effort to mitigate airway hyperreactivity [5]. In a DMBA treated oral mucosal carcinogenesis rat study model, SA when pre-administered was found to be anti-tumorigenic with lesions exhibiting only a keratotic effect and mild hyperplasia [6]. Along with the antitumorigenic and other multifarious benefits of SA, it was also shown to provide a protective effect on Acetaminophen induced damage in wistar rats [7]. SA was known to reduce oxidative damage in the liver of diabetic rats. It has a proven anti-inflammatory effect against denaturation of proteins, proteinase activity [8]. Therefore, it is of interest to document the molecular docking analysis of syringic acid with proteins in inflammatory cascade such as TNF α, NFκB, P50, P65 and IKB for further consideration in drug discovery.

## Materials and Methods:

### Preparation of ligands:

PubChem compound records were evaluated to confirm the structure of ligand Syringic Acid used for the present study. The structure was pictured using the tools available in ChemSketch (ACD/ChemSketch version 11.0. It was also instrumental in generating the 2D structure, to refine the 2D structure of the ligand, to perform 3D optimization and visualization of the 3D structure of the ligand.

### Preparation of target protein:

The structure of Tumor necrosis factor, P50, IKB, P65, NFKB evaluated using the X-ray diffraction method was retrieved from Protein Data Bank. Resolution of above 2.5 Angstrom was a mandate for selection and analysis. The pdb structures contributed to the atom coordinates in a flat file format. Heteroatoms and other bound residues were eliminated pdb files were recorded for the protein structures. The obtained pdb structures were 3D optimized prior to being subjected for docking.

### Docking simulations of target protein with syringic acid:

Argus Lab v 4.0.1 was used for docking. The binding sites in the target proteins were grouped and grid size was also calculated. The grid was prepared with 0.4-unit resolution. The docking was performed using Lamarckian Genetic Algorithm. The grid was centered on the ligand binding site of the target proteins for docking. Docking of the syringic acid with target proteins were performed using Argus Lab 4.0. Both the target protein and ligand were optimized for proper geometry, prior to docking. The target proteins along with syringic acid were simulated based on their optimized structures. Intermolecular flexible docking simulations were performed to yield docked conformations and energy values were calculated. Each docking run was repeated thrice to get the optimum result. The results of docking analysis were recorded as pdb files.

### Visualization:

Pymol, an open-source visualization tool in structural biology was used to produce high quality 3D images of these biological molecules and the docking simulations of target proteins. Interaction was measured by the binding energy of the best ligand pose measured in kcal/mol.

## Results:

### Syringic acid:

Figure 1A(see PDF) depicts the structure of Syringic acid as shown in the image. The syringic acid molecule will further be evaluated in interaction with various molecules of the inflammatory cascade.

### Inhibitor kappa B (ikb) - IKB binding site:

The molecule Syringic acid binds to the IKB at the IKB binding site as shown in (Figure 1B - see PDF) with 2 hydrogen bonds on same side, the bonds occur at chain D of the molecule with an energy expenditure of - 6.508 K cal. The Ikb h bond measures are of 3.99 Å and 3.04 Å depicting very close bonds with good bond strength.

### Nuclear Factor Kappa β (NFK β):

NFKB binds with Syringic acid very firmly at the binding site (Figure 1C - see PDF) with formation of 2 hydrogen bonds at either side of the structure at the P chain. The hydrogen bonds measure 2.62 Å or 2.71 Å respectively with very tight interaction being shown and strong specificity to the bonds. The energy expenditure was shown to be -6.892 Kcal.

### P50:

The interaction between P50 and syringic acid is shown here (Figure 1D - see PDF) with three hydrogen bonds at different sides at the B chain. The length of the hydrogen bonds is 2.42, 2.98 and 2.87 Å respectively. It shows strong binding.

### P65:

The interaction between P65 and Syringic acid is depicted here (Figure 1E - see PDF) with four hydrogen bonds with lengths being 2.83, 2.98, 3.01 and 2.72 Å respectively. The shorter the hydrogen bonds, much higher the interaction. Therefore the interaction is strong.

### Tumor Necrosis Factor-α:

Syringic acid binds with TNF alpha with 6 hydrogen bonds (Figure 1F - see PDF). Syringic acid strongly interacts with TNF alpha. Of all the molecules studied TNF alpha shows the strongest interaction with 6 hydrogen bonds. The measurements of these hydrogen bonds are 2.64, 2.86, 3.35, 2.83, 2.78, 2.72Å. The A chain of the molecule has been taken for interaction. The energy spent is -7.132 Kcal.

## Discussion:

The interactions of SA with TNF-α, P 50, IKB, P65 and NFKB were analyzed by docking method. This pathway was chosen because this was one of the most common pathways in inflammatory reactions and each of the above parameters are interlinked and therefore has a direct and/or indirect effect on the other as part of an inflammatory cascade.

### Tumour Necrosis Factor α (TNFα):

TNFα is an inflammatory cytokine produced predominantly during acute inflammation and kicks off a plethora of signalling events, leading to cell death by either necrosis or apoptosis. TNFα exerts many of its effects by binding, as a trimer, to TNFR‐1 or TNFR‐2, both cell membrane receptors [9]. A study on Salivary TNFα in periodontal disease with four study groups detected the highest TNFα levels in group 3, the diabetic group (diabetic status of more than 5 years) with pocket probing depth (PPD) ≥5 mm and clinical attachment loss (CAL) of ≥2 mm in comparison with the control group, the periodontal disease group without associated systemic diseases and the smoking group [10]. In comparison between 30 Oral Lichen Planus (OLP) and Oral Lichenoid Reaction (OLR) patients, it was found that the degranulated mast cells, ratio of degranulated mast cells and TNF-α was found to be elevated significantly in OLR than in OLP patients thus opening a potential of diagnostic marker to differentiate these entities [11]. TNF- α was considered as the crucial factor in the pathogenesis of many autoimmune and inflammatory diseases, such as Systemic Lupus Erythematosus, Rheumatoid Arthritis and Psoriasis [12,13]. TNFα has been implicated a key role in the pathogenesis of OLP. TNF-α has a marked effect on epithelial cells being cytotoxic at higher concentrations and antiproliferative at lower concentrations. The profile of surface molecules on certain key cells such as keratinocytes, Langerhans cells and endothelial cells was investigated in OLP and was found to support the activity of TNF- α in OLP [14]. Altered levels of various cytokines have been reported in Oral Potentially Malignant Disorders (OPMDs). TNF- α and IL-6 was evaluated in Oral Leukoplakia against controls and was found to reveal an increased expression of TNFαin association with Oral cancer and in Oral Potentially Malignant Disorders [15]. The concentration of TNFα had a bearing on its function. Low TNF-α could have an effect of diminishing the levels of ALT and AST in the plasma of TNF-αnull rats and promote the proliferation of hepatocyte cells. However, the levels of ALT and AST increased gradually with increasing TNF-α concentration after reaching the lowest value [16]. Theliver fibrosis resulting from chronic Carbon Tetra Chloride exposure was analyzed in a rat model and was found to be markedly dependent upon TNFα since there was almost a complete histological absence of fibrosis in TNFR-deficient mice [17]. Elevated TNF levels in alcoholic hepatitis patients play a crucial role in mediating hepatocyte damage and correlate inversely with patient survival [18]. In the docking study, TNFα was found to have the strongest bond with 6 hydrogen bonds. This was corroborated by various articles emphasizing the key role of TNFα in the various inflammatory pathways leading onto periodontal destruction, a biomarker of Oral cancer and OPMDs and other systemic diseases. Salivary TNFα can be used for predicting Oral Leukoplakia and Oral Squamous Cell Carcinoma (OSCC) [19]. TNFα can have a crucial role in hepatic fibrosis, alcoholic hepatitis as well as elevation of liver enzymes. Many molecular docking studies have depicted the crucial role of various elements in an emphatic manner [20,21-22].

### Nuclear Factor Kappa B (NF-κB):

Activation of the NF-κB is initiated by the signal-induced degradation of IκB proteins. This is mediated by IKB kinase (IKK). With the degradation of IκB, the NF-κB complex enters the nucleus where it turns on the expression of specific genes. The genes activated by NF-κB leads to the specified response which can vary from a cell survival response to a proliferation response. NF-κB has an auto regulatory effect in that it turns on expression of its own repressor, IκBα [23,24]. NF-κB family members play central roles in the interplay of genes involved in various biological phenomena for example, inflammatory response and/or Carcinogenesis [25]. NF-κB controls gene expression, including genes encoding for proinflammatory cytokines (e.g., interleukin 1[IL-1], IL-2, IL-6, IL-8,TNF-α, BAFF, and BLyS), chemokines (e.g., IL-8, MIP-1α, MCP1, RANTES, eotaxin, B-lymphocyte chemoattractant, and secondary lymphoid tissue chemokine), adhesion molecules (e.g., intercellular adhesion molecule 1, vascular cell adhesion molecule 1, and E-selection), inducible enzymes (e.g., COX-2 and inducible nitric oxide synthase), growth factors, some acute-phase proteins, and immune receptors, all mediators of inflammation [26,27]. P50 NF-kappaB subunit can have an ameliorative effecton the liver by interaction with TNF-α and the cascade of factors [28]. Several Pro-angiogenic and pro-inflammatory cytokines were evaluated in a study where in comparison of OPMDs and OSCC was done. The study suggested that the cytokines TNF-α & other cytokines dependent on NF-κB were elevated in the saliva of patients with OSSC and OPMDs as compared to controls. This can be used in therapeutic application [25]. Few studies documented the activation of NF-κB in diseased periodontal tissues [29,30]. NF-κB activation controls Matrix Metalloproteinase (MMP)and its production which is the key destructive enzyme in periodontal tissue destruction [31]. NF-κB mediates periodontal tissue remodelling by means of inducible forms of COX-2 and iNOS enzymes [32]. It also mediates neo- vascularization by mediation through Vascular Endothelial Growth Factor (VEGF) [33]. NF-κB, Toll like receptors and P53 was evaluated in Oral Lichenoid Diseases (OLD) revealing stronger immune reactivity for NF-κB and P53 in the intermediate layers of OLD than healthy controls [34]. NF-κB is thought to play a role in Oral Cancer progression and invasion. A combination of NFKB1or NFKBIA gene polymorphisms and environmental carcinogenesis may be related to an increased risk of oral cancer. Bone invasion by OSCC is a sequela of events controlled by a variety of molecules regulating interplay of factors aiding in OSCC proliferation and bone invasion. The therapeutic potential of NF-κB in inhibition of OSCC is being explored in depth [35]. Many inter-relationships exist between NF-κB, IKK and inflammatory mediators. Therefore, the NFKB- IKB Kinase complex is an area of further research for therapeutic intervention [36]. The cardioprotective ability of COMB [combination of Syringic acid (SA) and Reservatrol (RV)] is measured against Isoproterenol (ISO) induced cardiotoxicity in rats, a docking study measuredthe affinity to transcriptional NF- KB domains and found it to be strong [37].

### NF-κB, IκB, p50 and p65:

In mammals, the NF-κB/Rel family comprises five members with p50, p65 (Rel-A), and other proteins, forming inactive complexes with the inhibitory molecules called IκB proteins. Two pathways unique to the NF-κB/Rel family 1) the canonical pathway activated by pathogens and inflammatory mediators, and 2) the noncanonical pathway mostly activated by developmental events. The most abundant form of NF-κB activated by pathologic stimuli via the canonical pathway is the p65:p50 heterodimer [38]. NF-κB transcription complexes initiate several cellular responses through the canonical pathway and has been shown to be constitutively activated in some types of cancer cells. It has also been documented that activation of IKK-β than IKK-α promotes the primary pathway of proinflammatory genes. Many of the natural products and drugs that have been tested for anti-cancer and anti- inflammatory effects show an inhibitory effect on NF-κB and its homo or hetero dimmers [39]. Hodgkin/Reed Sternberg cells characteristic of Hodgkin's lymphoma shows evidence of constitutive activation of NF-κB [40]. The protein expression of NF-κB (p50/p65) was found to be increased in chronic periodontitis cases as opposed to aggressive periodontitis cases [41]. In our study, the effect of Syringic acid on these inflammatory markers was observed by the docking model. The marker with 4 hydrogen bonds and with strong affinity because of the shorter hydrogen bonds was P 65. The inflammatory marker with 3 short hydrogen bonds and therefore strong affinity was P50 followed by NF-κB with 2 hydrogen bonds and IKB.

## Conclusion:

We document the molecular docking analysis of syringic acid with proteins in inflammatory cascade such as TNF α, NFκB, P50, P65 and IKB for further consideration in drug discovery.
